# Diet Supplemented With Synthetic Carotenoids: Effects on Growth Performance and Biochemical and Immunological Parameters of Yellow Perch (*Perca flavescens*)

**DOI:** 10.3389/fphys.2019.01056

**Published:** 2019-08-21

**Authors:** Eman A. Abd El-Gawad, Han-Ping Wang, Hong Yao

**Affiliations:** ^1^Aquaculture Genetics and Breeding Laboratory, The Ohio State University South Centers, Piketon, OH, United States; ^2^Department of Aquatic Animals Diseases and Management, Faculty of Veterinary Medicine, Benha University, Toukh, Egypt

**Keywords:** digestive enzymes, antioxidant status, gene expression, carotenoids, yellow perch

## Abstract

The current study assessed the effect of dietary canthaxanthin and lycopene supplementation at different concentrations on growth performance and antioxidant status in yellow perch (*Perca flavescens*). In this regard, fish with initial weight (32 ± 1.0 g) were divided into five groups in triplicate, and fed on carotenoid-free diet (control), canthaxanthin (CTX) (50 and 100 mg/kg diet), and lycopene (200 and 400 mg/kg diet) for 60 days. Growth parameters and antioxidant enzymes were evaluated after 30 and 60 days post feeding. Tissue liver and intestine from six fish per treatment was collected for antioxidant and digestive enzymes analysis. The results revealed a significant increase (*P* < 0.05) of lipid content in the group fed lycopene at a dietary level 400 mg/kg for 60 days, compared to the control. Moreover, dietary carotenoids exhibited no significant effect on growth performance; this was evidenced by no significant up-regulation of growth hormone (*gh*) and insulin-like growth factor 1b (*igf-1b*) genes after 30 and 60 days post feeding. Intestinal lipase and trypsin activities were significantly improved with dietary lycopene especially at a dose of (400 mg/kg diet) for 60 days. Malondialdehyde (MDA) level in liver was also significantly decreased with dietary lycopene (400 mg/kg diet) for 60 days. Hepatic superoxide dismutase (SOD), catalase (CAT), and Glutathione peroxidase (GSH-Px) activities were significantly decreased with dietary CTX, especially at dose (100 mg/kg diet) and lycopene at a concentration of 200 and 400 mg/kg diet after 60 days feeding. Additionally, the immune-related gene interleukin-1 beta (*il-1b*) mRNA expression level revealed up-regulation in groups fed on CTX at different concentrations for 30 days, and fish fed lycopene at a concentration level 400 mg/kg diet for 60 days. The obtained results concluded that dietary supplementation of canthaxanthin and lycopene could enhance immune response and maintain antioxidants defense of fish. Therefore, it considered as a functional aquafeed ingredient for yellow perch.

## Introduction

Yellow perch *Perca flavescens*, are considered an important recreational food fish in North America (Brown and Barrows, 2002). Demand for yellow perch is high and varies with its relative availability and regional preference, however declining commercial harvest has created widespread interest in culturing yellow perch in the North Central Region (Brown et al., 1996). Unfortunately, environmental and husbandry stressors such as handling, capture, transport, and environmental quality under intensive aquaculture greatly influence the natural immune system of fish and may result in disease outbreak and, consequently, economic losses (Austin and Austin, 2016). Stress conditions increased the production of reactive oxygen species (ROS) resulting in oxidative stress, which affects fish immune response (Evans and Cooke, 2004). Many previous studies showed that antioxidant molecules such as vitamin C and E and carotenoids are modulators of the stress response in several fish species (Ortuno et al., 2003; Girao et al., 2012; Abd El-Gawad and Abdel Hamid, 2014; Sahin et al., 2014).

Carotenoids, belonging to family of fat-soluble pigments, have various sources including natural source (fungi, yeast and algae), animal source (crustacean), and synthetic source (lycopene, canthaxanthin, zeaxanthin, astaxanthin and β-carotene). They have been ascribed a wide range of biological functions in aquaculture including growth enhancement, improve skin coloration, antioxidant properties, precursors for vitamin A and transcription regulators, immune-system stimulants (Amar et al., 2004; Supamattaya et al., 2005; Zhang et al., 2013a, b, Sallam et al., 2017; Cheng et al., 2018). The physiological mechanism of carotenoids in preventing oxidative stress exerts in different ways as they can act as quenchers of singlet molecular oxygen or convert hydroperoxides into more stable compounds. Additionally, carotenoids can prevent formation of free radicals through the block of free radical oxidation reactions and inhibition of the autoxidation chain reaction (Galasso et al., 2017).

Lycopene is a red colored carotenoid present in tomatoes, watermelon, and carrots. Recently, it has attracted considerable attention as a chemoprotectant agent because of its highly antioxidant scavenging activity (Yonar, 2012; Ural, 2013; Sahin et al., 2014), thus preventing oxidative damage to cells and tissue, toxicity, and disease. Rao and Rao (2007) has been reported that lycopene exert its biological effects via different mechanisms that include gene function regulation, gap junction communication, hormone and immune modulation, carcinogen metabolism, and metabolic pathways involving phase II drug-metabolizing enzymes. Therefore, lycopene is considered as the most effective carotenoids used against biological ROS (Sahin et al., 2014). Canthaxanthin (β, β -carotene-4,4′-dione) is a keto-carotenoid generated naturally in a wide variety of algae (Hertzberg and Liaaen-Jensen, 1966). Canthaxanthin dietary inclusion in fish diet as a feed additive for enhancing growth and skin color of fish has been previously investigated (Storebakken and No, 1992; Kalinowski et al., 2005). As fish cannot synthetize their own carotenoids *de novo* (Gupta et al., 2007), hence, there is a need to incorporate these carotenoids in aquafeed as an essential micronutrient (Baker et al., 2002). To the best of our knowledge, no studies on the effect of these dietary carotenoids on digestive enzymes (lipase, amylase and trypsin enzymes) or immune related gene (*il-1b*) or growth related gene (*gh* and *igf-1b*) expression have been reported in fish. Therefore, the aim of the current study was to evaluate the effect of a diet supplemented with synthetic carotenoids (canthaxanthin and lycopene) on growth performance, immune response, and antioxidant status of yellow perch.

## Materials and Methods

### Experimental Diets

Canthaxanthin powder (Carophyll red 10%)^®^ was purchased from the eBay Company (CA, United States) and lycopene powder was obtained from the Kalyx Company (Camden, NY, United States). A commercial basal diet (AquaMax grower 400)^®^ was crushed and divided into five portions. The first portion (Diet 1, D1) was used as a control without additive. The second (Diet 2, D2) and third (Diet 3, D3) portions were mixed with canthaxanthin (CTX) at a concentration of (50 and 100 mg/kg diet), respectively, while the fourth (Diet 4, D4) and fifth (Diet 5, D5) portions were mixed with lycopene at a concentration of (200 and 400 mg/kg diet), respectively. The diets were reformed into pellets (2 × 3 mm) using an extruder (Brabender Plasti-Corder, Model PL 2000, South Hackensack, NJ, United States). Then pellets were dried in an oven for 24 h at 50°C to maintain the activity of CTX and lycopene in each diet. The dried pellets were kept in dark plastic packs and kept in a freezer at −20°C to avoid degradation of carotenoids and oxidation. The proximate analysis of experimental diets was carried out in Dairyland Laboratories, Inc., Arcadia, WI, United States. The proximate composition of the experimental and control diets are presented in Table 1.

**TABLE 1:** Proximate chemical analysis of experimental diets.

**Proximate analysis (%)**	**Diet 1**	**Diet 2**	**Diet 3**	**Diet 4**	**Diet 5**
Dry matter	95.25	95.71	95.28	95.06	94.45
Moisture	4.75	4.29	4.72	4.94	5.55
Crude protein	44.85	45.54	45.62	45.08	44.79
Crude lipid	11.71	11.88	11.73	11.68	11.34
Ash	9.02	9.02	9.12	8.91	8.90

### Fish and Experimental Design

Adult yellow perch were obtained from, and experiment was conducted in, the Ohio Centers for Aquaculture Research and Development, The Ohio State University, United States. Fish with (mean ± SE) 32 ± 1.0 g and 13.5 ± 0.6 cm were randomly divided into five groups in triplicate in round fiberglass tanks (60 L capacity); each replicate contained 18 fish. All groups were allowed to acclimate to the hatchery conditions for 2 weeks before starting the experiment. During the acclimation period, fish were hand fed commercial pellet (AquaMax 400)^®^ twice per day at 9:00 and 16:00 and tanks were continuously provided with natural ground water. Temperature was maintained at 15 ± 1.0°C and dissolved oxygen concentration at 6.8 ± 0.5 mg/L.

After acclimation, the first group was maintained as a control and fed with basal diet (D1). The second and third groups received a diet containing 50 (D2) and 100 (D3) mg/kg diet CTX, respectively, while the fourth and fifth groups were fed with lycopene at a concentration of 200 (D4) and 400 (D5) mg/kg diet, respectively. All experimental groups were fed by hand at a rate of 3% of their body weight. Feeding amount was divided into two equal parts twice per day (at 9:00 and 16:00) for 60 days. The rations were kept in small food containers at −20°C to prevent degradation or oxidation of lycopene or CTX. The amount of feed was adjusted every 2 weeks based on weight and the number of fish in each tank. All fiberglass tanks were supplied with throughflow well ground water and the photoperiod during the experimental period was adjusted for 12 h light: 12 h darkness. Fecal matter was siphoned out and 1/3 of the water was exchanged daily to maintain water quality parameters. The water parameters were monitored twice per day throughout the experiment according to the guidelines of Apha. (1998). The temperature ranged from 15.5 to 16.5°C, pH 6.8–7.2, and dissolved oxygen 6.9–8.5 mg/L. This study and all experimental procedures involving the care and use of animals were performed according to the protocol that was approved by The Ohio State University Institutional Animal Care and Use Committee.

### Collection of Samples

Fish were sampled at day 0 (initial), 30, and 60. They were firstly anesthetized using MS222 (250 mg/L). The liver samples were quickly removed from six fish per dietary treatment (2 fish/replicate) at each sampling point and preserved in phosphate buffer saline with pH 7.4 at −80°C. Another piece from liver tissue was kept in RNAlater (Ambion, United States) and preserved at −80°C for later gene expression. Also, intestines of treated and control fish were taken, emptied, and washed with an ice-cold phosphate buffer (pH 7.4) two times. After that, a piece from the mid-part of intestine was cut and preserved in phosphate buffer saline at −80°C until assay of the digestive enzymes.

### Determination of Growth Performance and Physiological Indices

After 30 and 60 days post feeding, growth performance and physiological indices in experimental groups were assessed according to the following formula: Weight gain rate (WGR%) = [(final body weight- initial body weight)/initial body weight] × 100, specific growth rate (SGR) = [(Ln final body weight − Ln initial body weight)/experimental period (days)] × 100, Feed conversion ratio (FCR) = [amount of feed intake (g)/weight gain]; Condition factor (CF) = [body weight (g)/body length (cm)^3^ × 100]; Hepatosomatic Index (HSI) = [weight of liver (g)/total body weight (g) × 100].

### Analysis of Body Proximate Composition

At the end of the experimental period, three samples of fish per group were randomly collected and kept at −20°C for body proximate composition analysis (moisture, crude protein, crude lipid and ash). Analysis of body proximate was carried out in Dairyland Laboratories, Inc., Arcadia, WI, United States. Moisture content was determined by drying at 105°C and ash content was measured by combustion at 550°C. Crude protein was analyzed according to standard methods (Aoac., 2000). Crude lipid was assessed using a Foss Soxtec 2047 instrument with the use of petroleum ether.

### Determination of Lipid Peroxidation and Antioxidant Enzymes

The liver samples were rinsed with phosphate buffer saline to remove any red blood cells, and then homogenized in cooled 50 mM phosphate buffer saline containing 1 μm EDTA (pH 7.4) at a ratio 1:5 (w/v). The procedure was performed on crushed ice. The homogenates were centrifuged at 13,000 × *g* at 4°C for 15 min, and the resultant supernatants were separated and pooled together (*n* = 3) to reduce analytic error and stored at −80°C until analysis. The pooled liver samples were used to estimate lipid peroxidation and antioxidant enzymes.

Malondialdehyde (MDA) as indices of lipid peroxidation in liver tissue was measured using commercial kits (BioVision, United States). The assay depends on reaction between MDA in the sample with thiobarbituric acid (TBA) to generate the MDA-TBA adduct. In this assay, the samples were treated with 600 μl TBA and incubated at 95°C for 60 min then cooled in room temperature for 10 min. The obtained reaction mixture can be easily quantified colorimetrically at OD 532 nm. Antioxidant enzymes activity, superoxide dismutase activity (SOD), Catalase activity (CAT), and Glutathione peroxidase activity (GSH-Px) were assessed spectrophotometrically using (BioTek’s Epoch^TM^, United States) following the procedures of commercial kits purchased from (Cayman Chemical Company, United States).

Superoxide dismutase assay kit utilizes a tetrazolium salt for detection of superoxide radicals generated by xanthine oxidase and hypoxanthine. Standard SOD activity was measured using bovine erythrocyte SOD solution. The total reaction volume of 230 μl was composed of 200 μl tetrazolium salt solution, 10 μl sample, and 20 μl xanthine oxidase, which imitate the reaction. The SOD activity was assessed at 440 nm and expressed as units. One unit of SOD activity is defined as the amount needed to exhibit 50% dismutation of superoxide radical.

The assay of CAT activity is based on the reaction of the CAT enzyme with methanol in the presence of H_2_O_2_ forming formaldehyde. In this reaction, 20 μl of sample, 100 μl potassium phosphate buffer (pH 7.0), and 30 μl methanol were added to each 96 well plate. The reaction was initiated by adding 20 μl hydrogen peroxide for 20 min, then 30 μl potassium hydroxide was added to terminate the reaction. CAT activity was measured spectrophotometrically at 540 nm and calculated using the following equation [CAT activity = [(μM of sample/20 min × sample dilution)].

The principle of measuring GSH-Px activity depends on reduction of hydroperoxides by GSH-Px enzyme forming oxidized glutathione, which is recycled to its reduced state by glutathione reductase. This assay uses NADPH, glutathione, and glutathione reductase as a co-substrate mixture, and the reaction is started by adding cumene hydroperoxide. The absorbance was read once every minute at 340 nm, and GSH-Px activity was determined using the following formula [GSH-Px activity = [(ΔA340/min/(0.00373 μM^–1^) × 9.5 ml × sample dilution]. The rate of decrease in the A_340_ is directly proportional to the GSH-Px activity in the sample.

### Determination of Digestive Enzymes

Samples taken from intestine were homogenized in cooled 50 mM phosphate buffer saline containing 1 μm EDTA pH 7.4 at a ratio 1:5 (w/v) using a glass homogenizer. The homogenates were centrifuged at 13,000 × *g* at 4°C for 10 min, and the resultant supernatants were separated and pooled together (*n* = 3) then stored at −80°C until analysis.

Lipase activity was assessed using Sigma- Aldrich kit, United States. Briefly, test samples were prepared to a final volume of 50 μl with lipase assay buffer, and then 100 μl reaction mix (93 μl lipase assay buffer, 2 μl peroxidase substrate, 2 μl enzyme mix and 3 μl lipase substrate) was added to each reaction and mixed by pipetting. The plate was incubated at 37°C for 2–3 min and absorbance was measured at 570 nm every 5 min until the value of the most active sample is greater than the value of the highest standard. One unit of Lipase is the amount of enzyme that will generate 1.0 mmole of glycerol from triglycerides per minute at 37°C.

Amylase activity was quantified using a Sigma-Aldrich assay kit, which depends on cleaves of substrate ethylidene-pNP-G7 to *p*-nitrophenol by the amylase enzyme. In this regard, 10 μl of sample was added into a 96 well plate and the final volume was completed to 50 μl with amylase assay buffer. Later on, 100 μl of master reaction mix (50 μl amylase assay buffer and 50 μl amylase substrate) was added and mixed well. Absorbance was measured at 405 nm every 5 min at 25°C. Amylase activity was reported as mU/ml. One unit of amylase is defined as the amount of amylase that cleaves ethylidene-pNP-G7 to generate 1.0 μmole of *p*-nitrophenol per minute at 25°C.

Trypsin activity was determined according to the procedure of commercial kit (BioVision, United States). The samples were prepared at 50 μl with trypsin assay buffer in a 96 well plate, then 1 μl of 50× chymotrypsin inhibitor (TPCK) solution was added and incubated for 10 min at room temperature. After incubation, 50 μl of reaction mix (48 μl assay buffer and 2 μl trypsin Substrate) was added to each well containing samples, and incubated at 25°C while being protected from light. The absorbance was measured initially and after 1–2 h incubation at 405 nm (BioTek’s Epoch^TM^, United States). Trypsin activity was expressed as units; one unit is defined as the amount of trypsin that cleaves the substrate, yielding 1.0 μmol of *p*-nitroaniline (*p*-NA) per minute at 25°C.

### Gene Expression of Growth and Immune Related Gene

Hepatic gene expression of growth-related genes (growth hormone (*gh*) and insulin-like growth factor (*igf-1b*) mRNAs) and immune-related gene (interleukin 1b (*il-1b* mRNAs) were evaluated at day 0, 30, and 60.

Total RNA was extracted from the liver using RNeasy mini kits (Qiagen, United States). According to the manufacturer’s instructions, 20 μg liver tissue was homogenized with 350 μl buffer RLT and 3.5 μl β-mercaptoethanol, then centrifuged for 3 min at high speed. The supernatant was carefully removed and mixed well by pipetting with 350 μl of 70% ethanol. The sample (700 μl) was transferred to a RNeasy mini spin column and centrifuged at 13,000 × *g* for 30 s. Buffer RW1 (700 μl) was added to the spin column and centrifuged. After discarding flow through liquid, 500 μl buffer RPE was added to the samples twice, and then centrifuged for 30 s at 13,000 × *g*. The extracted RNA was re-suspended in RNAs dNase nuclease-free water (Ambion, United States). Total RNAs were quantified at 260/280 nm using a spectrostar nanodrop (BMG LABTECH Inc., Cary, NC, United States) to evaluate their concentration and purity, and also analyzed whether they were degraded on a Gel Doc^T*M*^ XR (Bio-Rad, United States) using 2% agarose gel electrophoresis. Total RNAs with clear ribosomal band and high RNA ratios (A_260_/A_280_ ≥1.8) were used for further experiments.

cDNA was synthesized using high capacity cDNA reverse transcription kits (Applied Biosystems, United States). The reaction was prepared by pipetting 10 μl of 2× reverse transcription master mix (RT) and 10 μl of RNA sample into each well of 96-well reaction plate, and mixed well by pipetting up and down twice. The plates were sealed and the reaction conditions were adjusted as follows: 25°C for 10 min, 37°C for 120 min, 85°C for 5 min, and 4°C thereafter. In order to adjust for the variation in quantity of input cDNA, the housekeeping gene β-actin was used as an internal control. Primers for RT-PCR were designed with reference to the known sequences of yellow perch (Table 2). Real-time quantitative PCR was performed in total volume 20 μl containing 10 μl SYBR Green Master Mix (Applied Biosystems, Warrington, United Kingdom), 1.5 μl cDNA, 1.5 μl each primer, and 5.5 μl RNAs dNase -free water. RT-PCR was performed as follows: 95°C for 10 min; 40 cycles of denaturizing at 95°C for 15 s, annealing/extension at 60°C for 60 s. We calculated the relative quantification of the target gene *il-1b*, *gh*, and *igf-1b* using the 2^–ΔΔ*CT*^ method and the value stood for an n-fold difference according to Zhang et al. (2013b).

**TABLE 2:** Nucleotide sequences of the primers for gene expression in yellow perch.

**Target gene**	**Accession number**	**Forward (5′ → 3′)**	**Reverse (5′– 3′)**
*gh*	AY007303	CGGAGGAGCAGCGTCAAC	CCCAGGACTCGACCAAACG
*igf-1b*	AY332492	CGCAGGGCACAAAGTGGAC	CCCAGTGTTGCCTCGACTTG
*il-1b*	GO656767.1	ATCTTGAGGTTGTGGAGGCA	GCACATTTCCACTGGCTTGT
*B -*actin	AY332493.2	GCCTCTCTGTCCACCTTCCA	GGGCCGGACTCATCGTACT

### Statistical Analysis

The SPSS 16.0 package program was used in analyses. One-way analysis of variance and Duncan test were used for the determination of the significance of differences among the groups at each feeding period. A value of *P* < 0.05 was considered statistically significant. Data was also analyzed by a General Linear Model (Two-way ANOVA) (SPSS 16.0 program) to test the interaction between treated groups and time of feeding on response variables. Statistical significance was considered at *P* < 0.05.

## Results

### Growth Performance

The results of the effects of CTX and lycopene on yellow perch growth performance are shown in Table 3. No mortality occurred throughout the experiment and fish exhibited good health conditions. Growth performance and physiological indices of yellow perch fed CTX or lycopene-supplemented diet recorded no differences (*P* > 0.05) from the control group fed a carotenoid-free diet. Nevertheless, higher growth rates were observed in groups fed a lycopene diet (400 mg/kg diet) for 60 days. In addition, there were no significant interaction (*P* > 0.05) between treated groups and time of feeding.

**TABLE 3:** Growth performance of yellow perch fed on canthaxanthin and lycopene supplemented diets at different concentrations for 60 days.

**Groups^∗^**	**IBW (g)**	**FBW (g)**	**WGR (%)**	**FCR**	**SGR (%)**	**CF**	**HSI**
**For 30 days**							
G1	31.51 ± 1.63	49.89 ± 1.87	59.06 ± 9.02	1.61 ± 0.28	0.77 ± 0.1	1.54 ± 0.05	3.03 ± 0.47
G2	32.26 ± 0.94	48.42 ± 1.34	50.20 ± 3.35	1.81 ± 0.13	0.68 ± 0.04	1.52 ± 0.02	2.22 ± 0.49
G3	32.9 ± 0.83	50.41 ± 1.19	53.25 ± 0.92	1.69 ± 0.03	0.71 ± 0.01	1.46 ± 0.04	2.6 ± 0.43
G4	33.68 ± 0.64	52.44 ± 2.04	55.6 ± 3.68	1.63 ± 0.11	0.74 ± 0.04	1.51 ± 0.01	2.49 ± 0.29
G5	32.71 ± 0.25	52.33 ± 1.41	59.94 ± 3.28	1.51 ± 0.08	0.78 ± 0.03	1.46 ± 0.01	3.16 ± 0.23
**For 60 days**							
G1	31.51 ± 1.63	73.71 ± 0.99^ab^	135.0 ± 10.82^ab^	1.35 ± 0.12^ab^	1.42 ± 0.08^ab^	1.38 ± 0.03	1.91 ± 0.68
G2	32.26 ± 0.94	69.6 ± 2.18^b^	116.0 ± 7.78^ab^	1.57 ± 0.11^ab^	1.28 ± 0.06^ab^	1.3 ± 0.02	1.71 ± 0.55
G3	32.9 ± 0.83	68.92 ± 2.29^b^	109.46 ± 3.81^b^	1.65 ± 0.06^ab^	1.23 ± 0.03^b^	1.45 ± 0.1	2.66 ± 0.49
G4	33.68 ± 0.64	73.37 ± 1.25^ab^	118.11 ± 7.7^ab^	1.54 ± 0.1^ab^	1.29 ± 0.06^ab^	1.43 ± 0.07	2.05 ± 0.45
G5	32.71 ± 0.25	78.36 ± 2.22^a^	139.66 ± 7.86^a^	1.3 ± 0.07^b^	1.46 ± 0.06^a^	1.30 ± 0.03	2.56 ± 0.36
*Two-way ANOVA*							
Groups		*	*	*ns*	*	*ns*	*ns*
Time		*	*	*	*	*	*ns*
Interaction		*ns*	*ns*	*ns*	*ns*	*ns*	*ns*

*Values are mean (*n* = 3 replicate) ± SE. Mean values in the same column for each parameter with no letters or same letter are non-significant (*P* > 0.05). ^∗^Group 1, control; Group 2, canthaxanthin (50 mg/kg diet); Group 3, canthaxanthin (100 mg/kg diet); Group 4, lycopene (200 mg/kg diet); Group 5, lycopene (400 mg/kg diet).*

### Body Composition

There was no significant (*P* > 0.05) effect for dietary canthaxanthin or lycopene on body composition of fish compared to the control (Table 4). Fish fed lycopene at a dietary level of 400 mg/kg diet exhibited higher lipid content than fish fed the control diet.

**TABLE 4:** Body composition of yellow perch fed on canthaxanthin and lycopene supplemented diets at different concentrations for 60 days.

**Groups^∗^**	**Moisture**	**Crude protein**	**Crude lipid**	**Ash**
G1	72.85 ± 0.61	19.88 ± 0.84	4.09 ± 0.17^bc^	4.38 ± 0.86
G2	71.63 ± 0.93	19.22 ± 0.88	3.86 ± 0.30^c^	4.68 ± 0.32
G3	70.72 ± 1.23	19.86 ± 0.98	4.52 ± 0.09^abc^	5.31 ± 0.91
G4	73.07 ± 0.86	19.87 ± 0.72	5.42 ± 0.34^ab^	3.99 ± 0.21
G5	72.31 ± 1.18	20.02 ± 1.16	5.86 ± 0.83^a^	4.18 ± 0.76

*Values are mean (*n* = 3) ± SE. Mean values in the same column for each parameter with no letters or same letter are non-significant (*P* > 0.05). ^∗^G1, control; G2, canthaxanthin (50 mg/kg diet); G3, canthaxanthin (100 mg/kg diet); G4, lycopene (200 mg/kg diet); G5, lycopene (400 mg/kg diet).*

### Antioxidant Enzymes Activity and MDA Level

**FIGURE 1:**
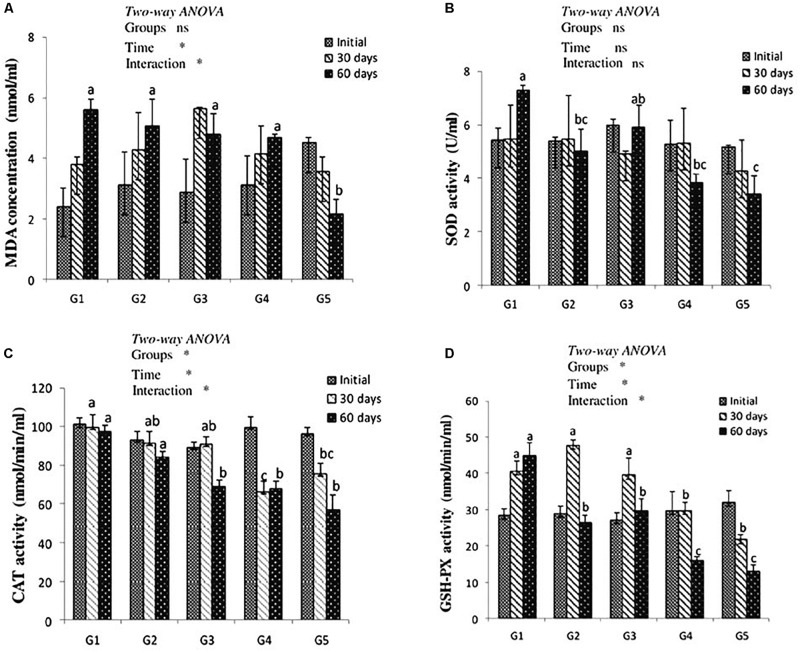
MDA level **(A)** and antioxidant of SOD, CAT, and GSH-Px activities (**B–D**, respectively) in the liver of yellow perch fed canthaxanthin and lycopene supplemented diets at 0, 30, and 60 days feeding. G1 fed with commercial basal diet (control). G2 and G3 fed with commercial basal diet containing 50 and 100 mg canthaxanthin/kg diet, respectively. G4 and G5 fed with commercial basal diet containing 200 and 400 mg lycopene/kg diet, respectively. Mean ± SE (*n* = 3). Means with no letter or with same letter indicate no significant differences (*P* > 0.05).

After 30 days of feeding, liver MDA level showed no significant difference among treated groups when compared to the control. After 60 days of feeding, MDA level was significantly decreased in yellow perch fed lycopene at a concentration of 400 mg/kg diet compared to other treated and control groups. MDA level was significantly (*P* < 0.05) affected by the interaction between experimentally treated groups and feeding time (Figure 1A).

The antioxidant enzymes activity is presented in Figures 1B–D. Dietary CTX and lycopene had no significant effect on SOD activity of yellow perch after 30 days of feeding. After 60 days of feeding, groups that received CTX at a concentration 50 mg/kg diet and lycopene at a dose of 200 and 400 mg/diet recorded a significant decrease of SOD activity. Yellow perch fed a diet supplemented with CTX at a concentration of 100 mg/kg revealed a significant decrease of CAT activity after 60 days of feeding. The incorporation of lycopene significantly decreased CAT activity throughout the experimental period. Liver GSH-Px activity of yellow perch showed a significant decrease with dietary lycopene at both concentrations during the experimental period, while fish treated with 50 and 100 mg CTX/kg diet exhibited a significant decrease in GSH-Px activity after 60 days of feeding. The interaction between feeding time and groups was significantly (*P* < 0.05) observed with CAT and GSH-Px activities, while SOD activity showed no significant (*P* > 0.05) effect.

### Digestive Enzymes Activity

**FIGURE 2:**
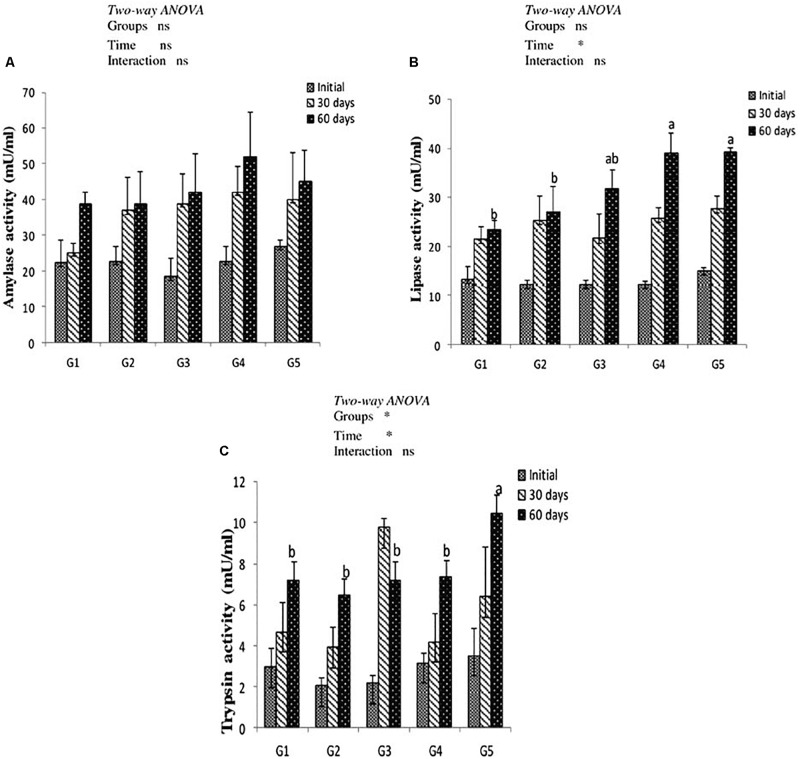
Digestive enzymes activity of amylase **(A)**, lipase **(B)**, and trypsin **(C)** in the intestine of yellow perch fed canthaxanthin and lycopene supplemented diets at 0, 30, and 60 days feeding. G1 fed with commercial basal diet (control). G2 and G3 fed with commercial basal diet containing 50 and 100 mg canthaxanthin/kg diet, respectively. G4 and G5 fed with commercial basal diet containing 200 and 400 mg lycopene/kg diet, respectively. Mean ± SE (*n* = 3). Means with no letter or with same letter indicate no significant differences (*P* > 0.05).

Results of digestive enzymes activities are shown in Figure 2. Amylase activity revealed no significant difference in fish fed CTX or a lycopene-supplemented diet for 30 and 60 days when compared with the control. Lipase enzyme activity recorded significant increases (*P* < 0.05) with dietary lycopene at a concentration of 200 and 400 mg/kg diet after 60 days of feeding. In addition, trypsin activity significantly increased with lycopene dietary supplementation at a concentration of 400 mg/kg diet for 60 days of feeding. Amylase, lipase, and trypsin enzymes activity were not significantly affected by the interaction between groups and feeding time.

### Gene Expression

**FIGURE 3:**
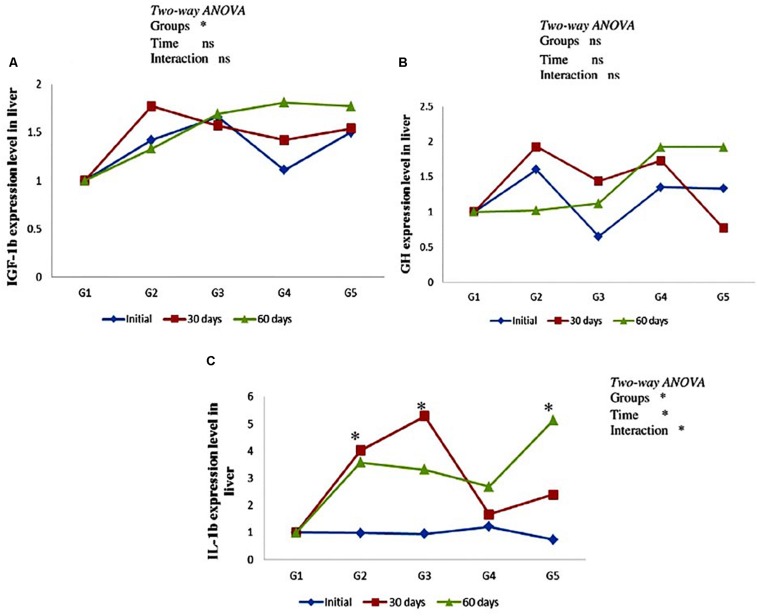
Genes expression level of hepatic *igf-1b* **(A)**, *gh* **(B)**, and *il-1b* **(C)** in the yellow perch fed canthaxanthin and lycopene supplemented diets at 0, 30, and 60 days feeding. G1 fed with commercial basal diet (control). G2 and G3 fed with commercial basal diet containing 50 and 100 mg canthaxanthin/kg diet, respectively. G4 and G5 fed with commercial basal diet containing 200 and 400 mg lycopene/kg diet, respectively. Data are mean (*n* = 3) ± SE. Values with asterisk (^∗^) are significantly different (*P* < 0.05).

As can be seen from Figure 3, there was no significant effect on hepatic *gh and igf-1b* mRNA expression level in yellow perch along the entire period of the experiment (Figures 3A,B). In addition, non-significant (*P* > 0.05) interaction between carotenoids treated groups and feeding time was observed. Immune-related gene *il-1b* mRNA expression level revealed up-regulation in groups fed on CTX at different concentrations for 30 days and fish fed lycopene at a concentration level 400 mg/kg diet for 60 days. Moreover, *il-1b* was significantly affected by the interaction between treated groups and feeding time (Figure 3C).

## Discussion

In the aquaculture industry, carotenoids are commonly incorporated in farmed fish and shellfish diets as pigmentation source to provide desirable coloration to these cultured organisms (Kalinowski et al., 2005; Kop and Durmaz, 2008; Li et al., 2017). It also has a positive effect on fish reproduction (Vassallo-Agius et al., 2001), antioxidant (Sahin et al., 2014), and immune systems. In addition, the ameliorative effect of synthetic carotenoids against stress such as pesticides exposure (Yonar and Sakin, 2011); antibacterial drug stress (Yonar, 2012); confinement (Girao et al., 2012) and high stocking density (Sahin et al., 2014) have been studied.

In the present study, dietary canthaxanthin and lycopene had no significant effect on the proximate composition of yellow perch compared to the control. This result comes in accordance with rainbow trout, *Oncorhynchus mykiss* that were fed astaxanthin for 60 days (Zhang et al., 2013a) and for 10 weeks (Rahman et al., 2016). Moreover, Zhang et al. (2013b) recorded that Pacific white shrimp, *Litopenaeus vannamei* fed astaxanthin supplemented diet revealed no significant difference in body composition. Carotenoids are lipid-soluble compounds and their absorption into enterocytes occur by passive diffusion or receptor-mediated transport after emulsification into mixed micelles which formed from bile salts, phospholipids, cholesterol, and lipolytic products such as free fatty acids, monoacylglycerols and lysophospholipids (Chimsung et al., 2014). However, micronutrient molecular structure, pH and bile lipid concentration, and the presence of a minimal amount of dietary fat can affect its transfer to mixed micelles (Tyssandier et al., 2001). In this sense, the physiological response of yellow perch growth with dietary lycopene at a level 400 mg/kg diet was better than other treated groups as a result of higher lipid content which might eventually regulate its uptake by intestinal mucosal cells and transportation processes (Castenmiller and West, 1998). In the same manner, Christiansen et al. (1995) recorded high lipid content in Atlantic salmon fed diets containing astaxanthin for 11 weeks. Therefore, the lipid content of a fish indicates the excess energy available for maintenance of growth (Tocher, 2003).

Dietary supplementation of canthaxanthin and lycopene did not affect growth performance, feed utilization, and survival of yellow perch. These findings were further supported by our results of the gene expression in the current study where the growth-related genes *gh* and *igf*-1b recoded no difference among treated and control groups. These results were similar with previous studies that recorded no significant effect for synthetic carotenoids in characins, *Hyphessobrycon callistus* (Wang et al., 2006), juvenile rainbow trout (Zhang et al., 2013a; Rahman et al., 2016), and Atlantic salmon, *Salmo salar* (Baker et al., 2002). The observation of no effect of dietary canthaxanthin on growth parameters was also recorded in red porgy, *Pagrus pagrus* (Kalinowski et al., 2005) and parrot cichlid (*Amphilophus citrinellus* × *Paraneetroplus synspilus*) (Li et al., 2017). Moreover, Nile tilapia, *Oreochromis niloticus* fed diet supplemented with lycopene 600 mg/kg for 60 days displayed no effect on weight gain, feed conversion ratio, and specific growth rate (Girao et al., 2012). Non-significant effect of carotenoids on yellow perch growth might be due to the lack of its absorption, which differed according to dietary levels and fish species, or might be due to the short period for dietary supplementation. Brown et al. (2009) stated that yellow perch native to North America has a relatively slow growth rate; this might be a possible explanation to our finding. In this study, yellow perch fed CTX-incorporated diets showed lower growth rate than the control group; it could be inferred that fish preferred the non-supplemented diet over those supplemented with carotenoids due to their flavor. Torrissen et al. (1990) recorded that higher inclusion of canthaxanthin with more than 50 mg/kg of feed in rainbow trout must be avoided. However, other studies observed that dietary carotenoids have a positive effect on nutrient utilization, specific growth, and improved weight gain (Amar et al., 2001; Zhang et al., 2013b; Alishahi et al., 2014; Cheng et al., 2018). This difference may be due to a difference of fish species, feeding behavior, as well as type, concentration, and duration of dietary carotenoids supplemented. Kop and Durmaz (2008) reported that the effectiveness of carotenoid is species-specific and that all fish species possess different pathways for the metabolism of carotenoids. Moreover, Kalinowski et al. (2005) suggested feeding fish with carotenoid-supplemented diets for a longer period to observe their significant role on growth.

Physiological indices such as condition factor, hepatosomatic, spleensomatic, and viscerosomatic are considered stress indicators in fish (Barton et al., 2002). All the experimental groups fed either canthaxanthin or lycopene in the current study recorded no significant difference in condition factor or hepatosomatic index, similarly as observed in rainbow trout that were fed astaxanthin (Zhang et al., 2013a). This indicated that dietary carotenoids had no detrimental effects on liver tissue or general fish health conditions.

Lipid peroxidation is considered to be a valuable indicator of cellular membrane damage caused by any stressors (Ferreira et al., 2005). The current study recorded no significant difference in liver MDA level among groups treated with canthaxanthin compared to the control. Meanwhile, the lycopene-treated group, at a dose 400 mg/kg diet, exhibited a significant decrease in MDA level after 60 days. A similar finding was previously reported by Yonar and Sakin (2011), Girao et al. (2012), Ural (2013), and Zhang et al. (2013a). During free radical scavenging, energy is transferred from singlet-oxygen to the lycopene molecule, which converts lycopene to the energy-rich triplet state. This indicates that the inclusion of lycopene decreased the MDA level, due to its free radical scavenging properties that delay lipid peroxidation by inhibiting the initiation or propagation phase of oxidizing chain reactions. Hence, it could protect tissue against the oxidation of lipids, proteins, and DNA (Matos et al., 2000; Wertz et al., 2004; Sahin et al., 2014).

Superoxide dismutase, CAT, and GSH-Px are considered the most important endogenous antioxidant enzymes required to convert superoxide radicals into hydrogen peroxide, and then into water and molecular oxygen. The activities of these enzymes reflect the ability of scavenging oxygen-free radicals, and the changes in redox status of a cell (Zhang et al., 2013a). Our results showed that canthaxanthin and lycopene fed fish had the lowest SOD activity after 60 days of feeding. These findings were in accordance with previous studies of other fish species fed carotenoid treated diets (Wang et al., 2006; Zhang et al., 2013a; Rahman et al., 2016). In addition, Yonar and Sakin (2011) recorded that carp, *Cyprinus carpio* received lycopene for 2 weeks and had no significant effect on hepatic SOD activity compared to control. Lygren et al. (1999) indicated that dietary supplementation of fat soluble antioxidants at high levels reduced the need for the production of endogenous antioxidant enzymes such as SOD and CAT, which are necessary for cell protection against reactive H_2_O_2_ and O_2_^–^. It seems that since dietary lycopene and canthaxanthin in yellow perch can already provide protection against ROS and hence there is less stimulation for the production of endogenous antioxidant enzymes.

Superoxide dismutase is part of the critical antioxidant enzyme system, which can convert the intracellular oxygen free radicals (O_2_–) into hydrogen peroxide (H_2_O_2_) and oxygen (O2). In addition, it plays an important role in the enhancement of phagocytic cell activity. The decrease of hepatic SOD activity in yellow perch that were fed carotenoid-supplemented diets in the current study could be attributed to the potential free radical scavenging activity of lycopene and canthaxanthin that effectively eliminate these radicals, or might be due to the inhibition of superoxide radical formation. The antioxidant mechanism of lycopene is a consequence of its chemical structure; it has 11 conjugated double bonds. Hence, due to its polyene structure, it can provide electrons to free radicals or attract unpaired electrons of free radicals, preventing lipid peroxidation and DNA damage. Canthaxanthin antioxidant mechanism occurs through capturing peroxyl-free radicals in its conjugated polyene system (Surai, 2012). Di Mascio et al. (1989) and Palozza et al. (2012) reported that lycopene is one of the most potent antioxidants, with a singlet-oxygen-quenching ability twice as high as that of β-carotene and 10 times higher than that of vitamin E.

Catalase rapidly catalyzes the decomposition of the damaging byproduct H_2_O_2_ into less reactive gaseous oxygen and water molecules, therefore, its high activity indicates that fish suffered from oxidative stress (Halliwell and Gutteridge, 2007). In the present study, dietary supplementation of lycopene and canthaxanthin significantly decreased hepatic CAT and GSH-Px activities in yellow perch after 60 days of feeding, indicating the strong capability of canthaxanthin and lycopene to quench singlet oxygen and subsequently maintain the redox status of cell. Similarly, significant decrease of CAT activity was also reported in Nile tilapia that were fed lycopene (Girao et al., 2012) and rainbow trout that were fed astaxanthin (Zhang et al., 2013a). Mourão et al. (2009) reported that exogenous antioxidants could decrease CAT activity. In this sense, fish supplemented with natural antioxidants could exhibit a reduction in the activity of CAT enzyme. Moreover, Halliwell and Gutteridge (2007) indicated that significant decrease of antioxidant activity could be argued to favorable maintenance of the redox state in the cell.

Glutathione peroxidase exists in blood, liver, mitochondria, and cytoplasm and plays a role in the removal reaction of H_2_O_2_; therefore, it is considered as one of the most important antioxidant defenses against oxygen toxicity inside the cells (Cohen and Doherty, 1987). The detoxifying process of H_2_O_2_ occurs through catalyzing the reduction in hydroperoxides using glutathione (GSH) producing glutathione disulfide (GSSG), which is reduced to GSH by glutathione reductase. The lower GSH-Px activity in yellow perch indicated that cell protection was activated as a result of dietary canthaxanthin and lycopene at different concentrations, especially for a long period of 60 days. It has been recorded that lycopene can upregulate the antioxidant electrophile/antioxidant response element, thereby stimulating the production of phase II detoxifying antioxidant enzymes that protect cells from reactive oxygen species and other electrophilic molecules (Ben-Dor et al., 2005; Palozza et al., 2012). Antioxidant defenses in fish are dependent on many factors such as age, feeding behavior, and nutritional factors. Radi et al. (1987), in a comparative study between different fish species, reported that carnivorous species had very low GSH-Px activity in the liver compared to other species. In this regard, yellow perch, as carnivore fish, might be exhibit low antioxidant activity. There is positive correlation between dietary supplementation with antioxidants and health status of fish (Küçükbay et al., 2009). Hence, the physiological response of antioxidant enzymes allow fish maintain their homeostasis, which necessary for physiological processes as growth, immunity and activation of stress response in fish (Trenzado et al., 2008).

Digestive enzyme activities among fish species vary by their age and feeding behavior (Péres et al., 1998). The growth of fish relies on nutrient utilization, which is reflected by the development of digestive organs and activities of intestinal enzymes (Yan and Qiu-Zhou, 2006). Therefore, the profile of digestive enzymes indicates the ability of a species to use different nutrients. To the best of our knowledge, there was no study on the effect of synthetic carotenoids on digestive enzymes activity in fish. In the current study, dietary canthaxanthin revealed no significant effect on digestive enzymes activity of yellow perch along the experimental period. This result was similar with findings recorded by Peixoto et al. (2016) in European sea bass (*Dicentrarchus labrax*). As a general assumption, amylase enzyme exhibit lower activity in carnivorous fish fed a diet of low carbohydrate level than omnivorous fish (Harpaz and Uni, 1999). This could be attributed to a non-significant difference of amylase enzyme in yellow perch, as it is a carnivore fish. Intestinal lipase and trypsin activities in the current study recorded a significant increase with dietary lycopene at a high concentration of 400 mg/kg diet for 60 days feeding. This finding was supported by Grosell et al. (2011), who reported that emulsification of fat and fat-soluble compounds into lipid micelles occurs in the small intestine and can increase efficiency of the lipase enzyme. Khani et al. (2017) revealed that natural microalgae *Chlorella vulgaris* supplementation significantly increases lipase enzyme activity in the intestine of koi, *Cyprinus carpio*. This could support our result, in which a significant increase of lipase activity was recorded in yellow perch that were fed diets containing lycopene, compared to canthaxanthin-treated groups. This increase might improve the digestion of fat, which could, in turn, explain the better growth of yellow perch with a lycopene-supplemented diet. The lower digestibility of canthaxanthin, when compared to lycopene, may be due to the presence of keto-hydrocarbon and esterification of the hydroxy-groups, which is the first step in the absorptive process (Young and Fox, 1936).

There was no available data on the dietary effect of synthetic carotenoids on gene expression of growth or immune-related genes in aquaculture. Therefore, this is considered to be the first study in yellow perch fed dietary carotenoids at different levels. Insulin-like growth factor Ib (*igf-1b*) is secreted by the liver and stimulated by *gh*, which in turn enhances growth rate (Suzuki et al., 2004). Growth-related genes [*gh* mRNA and *igf-1b* mRNA] expression level revealed no significant up-regulation in groups fed on canthaxanthin or lycopene for 60 days; this explains, to a certain extent, the non-significant effect on growth performance in these groups compared to the control. This could be attributed to the exposure of fish to acute handling stress, which can down-regulate the expressions of certain genes related to growth (Nakano et al., 2013). Insulin like growth factor-I was down-regulated in the liver of Nile tilapia fingerlings that were fed probiotics and prebiotics (Hassaan et al., 2015). Elabd et al. (2016) reported that incorporating *Astragalus membranaceus* and *Glycyrrhiza glabra* (liquorice) in the diet of yellow perch markedly up-regulated the expression of growth-related genes, insulin-like growth factor-1 (*igf-1*). Cytokines are regulatory proteins secreted by immune cells that initiate and regulate cellular function such as the immune response, inflammation, acute phase response, and tissue repair (Ollier, 2004). Interleukin-1b (*il-1b*) are synthesized by hepatocytes and their production is considered to be indicators of an inflammatory response (Secombes et al., 2001), and they can regulate the production of other cytokines. In our study, the *il-1b* gene revealed up-regulation in groups fed on canthaxanthin at different concentrations for 30 days, and fish that were fed lycopene at a concentration level of 400 mg/kg diet for 60 days, compared to the control. This gene has a crucial role in the host response during microbial invasion and tissue injury due to the enhancement of phagocytic activity, macrophage proliferation, lysozyme synthesis, and leukocyte migration (Magnadóttir et al., 2005). This result coincides with Giri et al. (2015) in *Labeo rohita* that were fed guava leaves and common carp, *Cyprinus carpio* fed *Rehmannia glutinosa* (Wang et al., 2015). Moreover, dietary Spirulina in common carp significantly increases the expression of interleukin-1b (Watanuki et al., 2006). On the other hand, Lee et al. (2003) recorded that astaxanthin inhibits the expression of inflammatory gene of tumor necrosis factor-α (*tnf-*α) and *il-1b* in mice.

It could be concluded that growth and feed utilization are not affected by dietary supplementation of canthaxanthin. However, lycopene-supplemented diets revealed a positive effect on digestive enzymes activity and growth of yellow perch. Both carotenoids have a protective effect by its antioxidant activity, and enhance yellow perch immunity through up-regulation of the immune-related gene (*il-1b*). In this sense, the inclusion of lycopene or canthaxanthin is recommended as dietary supplements in yellow perch for protection against oxidative stress and disease occurrence.

## Ethics Statement

This study and all experimental procedures involving the care and use of animals were performed according to the protocol that was approved by The Ohio State University Institutional Animal Care and Use Committee.

## Author Contributions

H-PW and EAE-G conceived the experiment. EAE-G conducted the experiment, data analysis, and drafted the manuscript. HY helped and verified the analytical methods. H-PW revised and finalized the manuscript. All authors discussed the results and contributed to the final manuscript.

## Conflict of Interest Statement

The authors declare that the research was conducted in the absence of any commercial or financial relationships that could be construed as a potential conflict of interest.
